# Association between School Membership and Substance Use among Adolescents

**DOI:** 10.3389/fpsyt.2018.00025

**Published:** 2018-02-08

**Authors:** Jorge Gaete, Graciela Rojas, Rosemarie Fritsch, Ricardo Araya

**Affiliations:** ^1^Department of Public Health and Epidemiology, Faculty of Medicine, Universidad de los Andes, Santiago, Chile; ^2^Centre for Global Mental Health, Department of Population Health, London School of Hygiene and Tropical Medicine, London, United Kingdom; ^3^Departamento de Psiquiatría y Salud Mental, Clínica Psiquiátrica Universitaria, Universidad de Chile, Santiago, Chile; ^4^Millennium Institute for Research in Depression and Personality, Santiago, Chile; ^5^Health Service and Population Research Department, Institute of Psychiatry, Psychology and Neuroscience, King’s College London, London, United Kingdom

**Keywords:** school membership, substance abuse, adolescents, depression, anxiety, mental health

## Abstract

**Background:**

Substance use among adolescents is a major problem worldwide, producing many health and economic consequences. Even though there are well-known personal, familial, and social factors associated with drug use, less is known about the effect of school-related factors. School membership is a recognized variable affecting academic performance among students; however, its effect on substance use is less understood.

**Aims:**

The primary aim of this study was to explore the association between school membership and cigarette, alcohol, and cannabis use among a representative sample of secondary students from municipal state-funded schools in Santiago of Chile, and secondly, to test the hypothesis that depressive or anxiety symptoms mediate this association.

**Methods:**

A total of 2,508 students from 22 state-funded schools in Santiago, Chile, answered a questionnaire. This instrument included an abbreviated version of the psychological sense of school membership (PSSM), questions regarding the use of alcohol, tobacco, and cannabis and scales of psychological functioning (depression, anxiety, self-concept, and problem-solving). The association analyses were performed using adjusted regression models for each outcome using all independent variables while controlling for gender and age. For the mediation effect, a combination of ordinary least square and logistic regression analyses was conducted.

**Results:**

There was an association between a strong PSSM and low risk for smoking (OR 0.57; 95% CI 0.46–0.72), drinking (0.65; 95% CI: 0.51–0.83), and cannabis use (0.52; 95% CI 0.37–0.74). We also found that depressive and anxiety symptoms do not fully mediate the association between school membership and any substance use, and 73% of this effect in the case of smoking, 80% in the case of drinking, and 78.5% in the case of cannabis use, was direct.

**Conclusion:**

This is the first study in Latin America exploring the association between school membership and substance use among secondary students. School membership seems to be an important and independent factor to be included in preventive interventions. Therefore, these results support future research aiming to test interventions at increasing the sense of school membership to prevent substance use among adolescents.

**Clinical Trial Registration:**

ISRCTN19466209. Retrospectively registered.

## Introduction

Substance use among adolescents is still a major problem worldwide producing many health and economic consequences (1). The most recent Chilean governmental report about substance use among 8th–12th graders says that the monthly prevalence of smoking, drinking, and cannabis use was 26.7, 35, and 18.8%, respectively. There has been no change in smoking and drinking behavior from the previous study, and there has been a clear increase in cannabis use over the last 10 years. These figures are very high when compared with other parts of the world. For example, in European secondary students, among all counties studied, it was found that an average of 21% of adolescents have used cigarettes; 48%, have used alcohol; and 7%, have used marihuana in the last 30 days (2). Comparing Chile with the rest of American countries, a recent report found that Chile has the highest prevalence of tobacco use in the last month and the prevalence of cannabis use in the last year of the continent (3) among secondary students. The same report shows that Chile is one of the 14 out of 29 countries studied in America, with a prevalence of alcohol use in the last month over 30%. However, when we observed the rate of progression between students in Year 8 to Year 12, Chile has the third leading place in Latin America (after Ecuador and Peru) increasing three times the prevalence during secondary school years (3).

Although several studies have explored the influence of personal, familial, school peer, and environmental factors on the use of substances of abuse among adolescents (4–6); the effect of school membership seems less well investigated. School membership is a concept of students’ perceptions about whether they feel accepted, respected, included, and supported by others in the school social environment (7). It is also defined as the perception of social bonds with other school members; something that will have implications in terms of self-identity and commitment among students of a specific school. It is recognized as an important determinant of school attendance, good behavior, effort in schoolwork (8), and having better academic results (9). The sense of school membership influences the way students become interested and engaged in everyday school activities (10, 11). It is also recognized as an important factor in school retention and participation of vulnerable students in their schools (12).

Few studies have investigated the association between school membership (e.g., feeling part of the school, feeling close to, and respected by the people at school) and substance use, and they have been conducted mostly in developed countries (6, 12–19). It seems that students who feel connected and part of their school are less likely to be engaged in smoking, heavy drinking, and cannabis use. On the contrary, students who feel less supported and respected by teachers and peers and less part of the school, may feel isolated, depressed or anxious resulting in a higher risk of using drugs, and to failing in school (12). Additionally, students who do not fell part of their schools are less likely to perceive substance use as a problem that may affect their future academic goals (16).

There are potential explanations for the association between school membership and substance use. As Fletcher et al. proposed (20), students who feel disengaged with school might use drugs as a way of bonding with other students who are also disconnected from the schools. Another reason (20) may be that the school environment may be perceived and felt too challenging (from school performance to group acceptance), and difficulties to perform well in this environment might generate frustration and emotional symptoms. Students might resort to using drugs to alleviate some of these symptoms or escape from the school pressure. To our knowledge, no previous studies from Latin America have explored this association and the potential mediation effect of depressive or anxiety symptoms.

The main purpose of this study is to explore the association between school membership and cigarette, alcohol, and cannabis use among a representative sample of secondary students from municipal state-funded schools in Santiago, Chile. A secondary purpose is to test the hypothesis that depressive or anxiety symptoms mediate this association.

## Materials and Methods

### Setting and Participants

The data presented here comes from the baseline assessment of the first large cluster randomized controlled trial in Latin America of a universal school-based aimed at reducing depressive symptoms among 9th graders from 22 municipal mixed-sex schools in Santiago, Chile. Informed consent was obtained from parents or main caregivers and assent from the students. All students at Year 9 were eligible to participate in the trial and all but two students provided data for this study. In this original trial, we found no evidence of the proposed intervention being better than usual care in any of the main outcomes. Further information about this trial can be found the publications of the protocol (21) and main results (22).

### Survey Administration

After obtaining authorization from the schools and consent from the parents or main caregivers, the evaluation research team approached all selected schools and classes and asked students for their assent. In April 2009, the baseline assessment was carried out using self-administered questionnaires, which took one standard academic hour (45 min) to be completed. The research team was well trained and they followed a strict protocol with actions to be implemented before (e.g., time and procedure to contact schools to arrange the date of assessment), during (e.g., how to answer questions from students), and after (e.g., how to enter data in pre-built datasets) the study. Teachers were asked to stay in the classroom to help if needed but were encouraged to stay away from pupils’ desks while students answered the questionnaires.

### Measures

All scales used in the questionnaire were pre-tested in four schools with similar background the academic year before the baseline assessment to make sure students could understand the questions and respond with consistency (21, 22).

#### Dependent Variables

We assessed three outcomes regarding substance use: smoking, drinking, and cannabis use. We used standard questions implemented in several school-based surveys internationally (23) and nationally (24) to allow comparisons. We asked for any use of cigarettes, drinking alcohol or smoking cannabis during the last 30 days prior to the survey (dichotomized into 0 “never” versus 1 “at least once”).

#### Independent Variables

##### School Membership

School membership was assessed using the eight-item abbreviated version of psychological sense of school membership (PSSM) (7), which has been used in other studies (25, 26). This scale measures if students feel valued, respected, and included in the school. Each statement may be answered using a scale from 1 “Not at all true” to 5 “Completely true.” The score is calculated summing up the answers for each statement and dividing by 8. A single latent factor was identified after performing an exploratory factor analysis, and the Cronbach alpha for this scale was 0.78. For the association analysis, we categorized students into tertiles: low (students with the lowest PSSM scores), medium (students in the middle third), and high (students with the highest PSSM scores) school membership. For the analysis of mediation effect, we use this score as a continuous variable.

##### Other Independent Variables

Gender and age.

Depression was assessed using the 21-item Beck Depressive Inventory, second version (BDI-II). This is a self-administered scale generating a total score from 0 to 63. It has been previously validated among Chilean adolescents (27). We dichotomized scores according to our own study of validation using the cut-off of 19/20 for girls and 13/14 for boys (28) to decide if the student was depressed. The Cronbach alpha for this scale was 0.89.

Anxiety was assessed using the Spanish version of the revised child anxiety and depression scale, but we included just the generalized anxiety, social anxiety, and panic sub-scales because depression was already assessed by the BDI-II and the separation anxiety subscale was less important for this age group. We also dichotomized around the median with a score ≥19 defined as high anxiety. The Cronbach alpha for this scale was 0.83.

Personal failure subscale of the children’s automatic thoughts scalewas used as a measure of self-concept. This 10-item scale gives a good idea of how well (or poorly) students think about themselves and detecting negative thinking (29). We dichotomized around the median with a score ≥8 defined as poor self-concept. The Cronbach alpha for this scale was 0.88.

Problem-solving skills were assessed using a modified version of the short version of the social problem-solving inventory revised. This scale assessed several aspects of problem-solving processes. Only 20 out of 25 items were used in this study because students had some problems with the understanding of some items. We dichotomized around the median with a score ≥45 defined as good problem-solving skill. The Cronbach alpha for this scale was 0.90.

### Ethics Approval and Consent to Participate

This study was approved by the local committee of ethics at the Hospital Clínico Universidad de Chile (No. 179; June 30, 2008), and informed and written consent from parents or main caregivers and informed and written assent from students were required to participate.

### Statistical Analysis

We performed a complete case analysis. First, we examined univariable associations between each independent variable and each outcome using logistic regressions. Then we performed adjusted regression models for each outcome using all independent variables, while also controlling for gender and age.

To explore the mediation effects, we followed guidance on mediation analysis with dichotomous data (30). In view that we had dichotomous dependent variables (smoking, drinking, or cannabis use), continuous independent variables (sense of school membership score), and binary variables as possibly mediating the effect on outcomes, we opted to use a combination of ordinary least square and logistic regression analyses to determine the indirect effect.

All analyses were carried out using STATA version 13.0.

## Results

### Descriptive Statistics

A total of 2,508 students participated in this study. Missing data for the baseline was small (9.4%). The mean age was 14.5 years old (range: 12–189 and 44.5% corresponded to girls).

A 38.7% of students smoked cigarettes, a 27.5% drunk alcohol, and a 13.8% used cannabis in the last 30 days.

The mean of school membership score was 3.7 (SD = 0.7).

Almost one in three students had a BDI-II score over the cut-off to be considered depressed. A high score of anxiety (≥19) was presented in 53.5% of students and also, more than half of students had poor self-concept. A 52.5% had a score under the category of good problem-solving (see Table 1).

**Table 1:** Descriptive characteristics, overall and by gender.

	Overall
Variable	*n* = 2,508
Age (12–18), mean (SD)	14.53 (0.89)
Smoking (last 30 days)	970 (38.74)
Drinking (last 30 days)	687 (27.47)
Binge drinking (last 30 days)	482 (19.29)
Cannabis use (last 30 days)	345 (13.80)
School Membership, mean (SD)	3.67 (0.72)
Low-school membership, mean (SD)	2.90 (0.43)
Medium school membership, mean (SD)	3.75 (0.18)
High-school membership, mean (SD)	4.49 (0.29)
BDI-II, mean (SD)	13.43 (10.24)
Depressed (≥20 in girls; ≥14 in boys)	777 (30.98)
RCADS, mean (SD)	19.69 (8.54)
High anxiety (≥19)	1302 (53.54)
Self-concept, mean (SD)	10.09 (7.91)
Poor self-concept (≥8)	1,351 (54.76)
Problem-solving skill, mean (SD)	44.82 (12.82)
Good problem-solving skill (≥45)	1,269 (52.46)

### Unadjusted Analysis

In Table 2, we can see that a higher sense of school membership is associated with a lower proportion of consumption of all drugs during the last month. When we observe the unadjusted results, it is possible to see the protective association of school membership: the higher the score the lower the likelihood of being a smoker, drinker, or marijuana user.

**Table 2:** Distribution of substance use behavior according to school membership.

School membership level	Smoking, *n* = 2,471	Drinking, *n* = 2,468	Cannabis use, *n* = 2,469

	*n*(%)	*n*(%)	*n*(%)
Low	419 (47.89)	308 (35.16)	160 (18.29)
Medium	309 (37.14)	208 (25.06)	113 (13.60)
High	227 (29.71)	163 (21.39)	64 (8.39)

χ^2^ (df), *p*	58.0178 (2), *p* = 0.000	42.5085 (2), *p* = 0.000	33.8809 (2), *p* = 0.000

### Adjusted Analysis

Students who have a higher level of school membership have a lower likelihood of being smokers (OR 0.57; 95% CI 0.46–0.72). We can notice that is a trend in this protective effect. Other factors associated with being a smoker were age, being girl, having more depressive symptoms, and poor self-concept (see Table 3).

**Table 3:** Unadjusted and adjusted associations of predictor variables with smoking, drinking, and cannabis use among Chilean adolescents.

	Smoking		Drinking		Cannabis use	
	Unadjusted	Adjusted	Unadjusted	Adjusted	Unadjusted	Adjusted
			
Variable	OR (95% CI)	OR (95% CI)	OR (95% CI)	OR (95% CI)	OR (95% CI)	OR (95% CI)
School membership						
Low	1	1	1	1	1	1
Medium	0.64 (0.53–0.78)**	0.73 (0.59–0.91)*	0.62 (0.50–0.76)**	0.72 (0.57–0.90)*	0.70 (0.54–0.91)*	0.77 (0.57–1.02)
High	0.46 (0.38–0.56)**	0.57 (0.46–0.72)**	0.50 (0.40–0.63)**	0.65 (0.51–0.83)**	0.41 (0.30–0.56)**	0.52 (0.37–0.74)**
Age	1.86 (1.69–2.05)**	1.84 (1.66–2.05)**	1.66 (1.50–1.83)**	1.57 (1.42–1.75)**	2.06 (1.81–2.34)**	2.00 (1.75–2.29)**
Gender						
Boys	1	1	1	1	1	1
Girls	1.48 (1.26–1.74)**	1.46 (1.21–1.76)**	1.02 (0.86–1.22)	1.00 (0.83–1.22)	1.30 (1.04–1.63)*	1.33 (1.02–1.72)*
Depressed	2.11 (1.78–2.51)**	1.52 (1.22–1.89)**	1.82 (1.52–2.20)**	1.43 (1.13–1.79)*	1.99 (1.58–2.51)**	1.29 (0.97–1.72)
High anxiety	1.30 (1.10–1.53)*	0.87 (0.71–1.06)	1.11 (0.93–1.33)	0.84 (0.68–1.04)	1.22 (0.96–1.53)	0.91 (0.69–1.21)
Poor self-concept	1.97 (1.67–2.33)**	1.47 (1.19–1.841)**	1.56 (1.30–1.87)**	1.28 (0.02–1.60)*	1.86 (1.46–2.37)	1.43 (1.06–1.93)*
Good problem-solving skill	0.79 (0.67–0.93)*	1.00 (0.83–1.21)	0.87 (0.73–1.04)	1.08 (0.89–1.32)	0.68 (0.54–0.85)*	0.84 (0.65–1.09)

*p ≤ 0.05.

**p ≤ 0.001.

Regarding drinking, we also found a strong reduction in the probability of drinking in the last month among those students who had a high sense of school membership (OR 0.65; 95% CI 0.51–0.83). Other variables associated with drinking were age, being depressed and having a poor self-concept (see Table 3).

Finally, a high level of school membership was also associated with lower risk of using cannabis during the last month (OR 0.52; 95% CI 0.37–0.74). Age, being girl, and poor self-concept were also associated with cannabis use during the last month (see Table 3).

**Figure 1:**
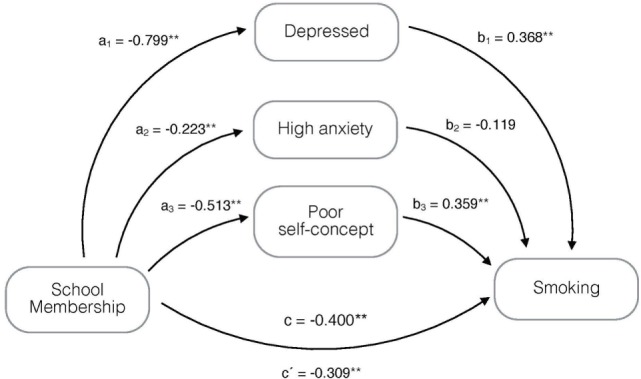
Mediation model, adjusted for age and gender, on the association of school membership with smoking and possible mediation by being depressed, having high anxiety and poor self-concept. *p < 0.05, **p ≤ 0.001; a_1_–a_3_, b_1_–b_3_, c = regression coefficients; and c′ = regression coefficient between the independent variable and dependent variable adjusted by the mediating effect.

Exploring the mediation effect, we found that being depressed and having a poor self-concept mediated significantly the effect of school membership on smoking (Figure 1). Approximately a 27% of the effect on school membership is mediated by being depressed and having a poor self-concept. However, a 73% of the total effect was *direct* and significant.

**Figure 2:**
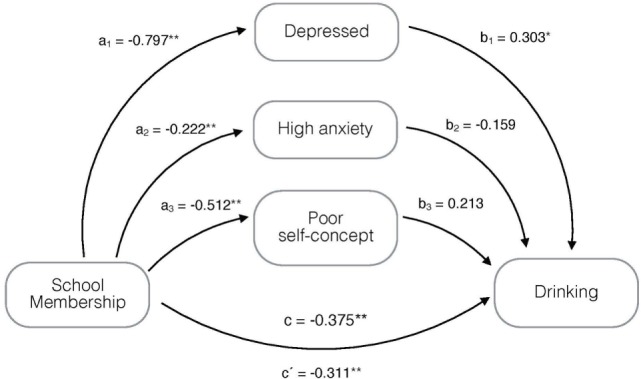
Mediation model, adjusted for age and gender, on the association of school membership with drinking mediated by being depressed, having high anxiety and poor self-concept. *p < 0.05, **p ≤ 0.001; a_1_–a_3_, b_1_–b_3_, c = regression coefficients; and c′ = regression coefficient between the independent variable and dependent variable adjusted by the mediating effect.

For drinking behavior (Figure 2), being depressed mediated part of the effect of school membership on drinking. This mediation reached a significance of up to 20% of the effect and approximately 80% was *direct* and significant.

**Figure 3:**
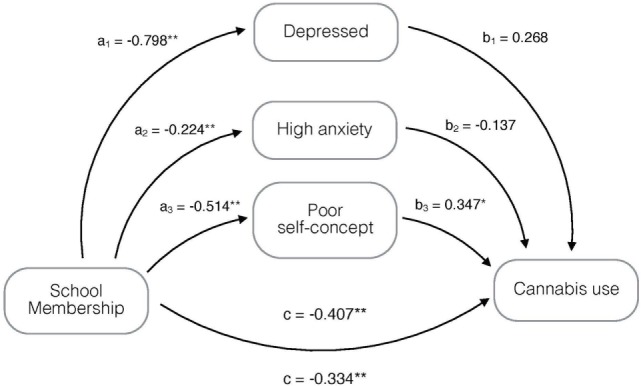
Mediation model, adjusted for age and gender, on the association of school membership with cannabis use mediated by being depressed, having high anxiety and poor self-concept. *p < 0.05, **p ≤ 0.001; a_1_–a_3_, b_1_–b_3_, c = regression coefficients; and c′ = regression coefficient between the independent variable and dependent variable adjusted by the mediating effect.

The association between school membership and using cannabis during the last month was significantly mediated by having a poor self-concept (21.5%) (Figure 3), and 78.5% of the variance of the association between school membership and cannabis use was *direct* and significant.

## Discussion

The use of cigarettes, alcohol, and cannabis during the last month is highly prevalent among Chilean adolescents. When comparing our prevalence results with those from others studies we have to be cautious because substance use is highly determined by age. This is because substance use increases gradually as adolescents get older and different countries have different laws regulating the legal age to purchase cigarettes, drink alcohol or to purchase/use cannabis, which ultimately seems to influence adolescent behavior (31). The mean age of our sample was 14.5 years old and results from a recent national survey in New Zealand among a similar population found that 11.4% of students smoked cigarettes and 10.1% smoked cannabis in the last month (32). Prevalence for use of all drugs was much lower than in Chile. The Monitoring the Future surveys in the United States include students from 8th, 10th, and 12th grades making it less comparable with our study. However, in 2014, among 8th and 10th graders, the prevalence of smoking cigarettes, alcohol use, and cannabis use in the last month ranged between 4 and 7.2%, 9 and 23.5%, and 6.5 and 16.6%, respectively (33). Therefore, smoking and drinking among ninth grade Chilean students is higher than in American students in 10th grade. Additionally, the trends in cannabis use among Chilean students are changing rapidly, increasing in prevalence at the highest rate since the start of data collection in Chile (24). The last national report of substance use in English adolescents between 14 and 15 years old showed that 21 and 40%, respectively, had their last alcoholic drink in the last month; while 5–10% of students 14 and 15 years old, respectively, took cannabis in the last month (34). Taking all of this into account, our results show that substance use in Chile is an urgent problem and resources should be given to better understand the causes of the problem and to the implementation of effective measures to reduce its use.

Depression and anxiety have been found associated with low-academic performance and high-school dropout among adolescents (35), indicating that students with psychological distress may be less motivated and involved with their schools. Additionally, adolescents with anxiety and depressive symptoms are more likely to be involved in drug use. There is a clear association between depression and anxiety and substance use among adolescents, but it seems to go in both directions (36–39). Some studies show that depressive symptoms are present before the experience of use of alcohol and drugs (40, 41), and others show that alcohol and drug use are risk factors for depression (42–44). On the other hand, students with low-school connectedness also have a higher risk for substance use (45, 46).

This study adds evidence to the role of anxiety and depressive symptoms in the association between school membership and substance use among adolescents. We found that school membership appears to have a large impact on reducing the risks of substance use behaviors, even after controlling for important individual confounders such as age, gender, or depressive symptoms. Therefore, school membership had an independent effect on drug use that seems to not be mediated by personal negative feelings such as depression or poor self-concept. School membership, or other related concepts such as connectedness, attachment, engagement, or bonding, has been considered an important factor influencing academic performance among students. In recent years, studies have related it to mental health and substance use. For instance, Bond et al., using a longitudinal design, found that those students with low-school connectedness at Year 8 had increased risk of anxiety/depressive symptoms, regular smoking, drinking, and use of cannabis in later years (12). However, this research used a multidimensional scale exploring school connectedness, with few items related to sense of school belonging. School connectedness/bonding should be studied taking into account the independent effect of each dimension. One example of this attempt is our recent study, where we explored the independent association of school commitment and school attachment with smoking, and we found that students who committed to their school activities were less likely to smoke (47). However, school attachment, the emotional bonds to the school, was not associated with smoking risk. In the present study, we have only explored the association between sense of school membership and substance use.

Most of the effect of school membership over smoking, drinking, and cannabis use is direct. However, we found some evidence that part of the effect was due to being depressed for the case of smoking and drinking. Additionally, having a poor self-concept accounted for part of the effect for smoking and was the only factor accounting for cannabis use. Mediator variables are not the same for different substances. However, we could think that students disconnected from their schools have some negative feelings (being depressed and poor self-concept) that, partially, may influence their use of different substances. We still need to study what other factors are mediating the effect of school membership and how this association and mediating effects change over time using longitudinal study designs.

We think that our findings support the idea of using school-based interventions that promote a sense of school belonging to prevent substance use (45). For instance, some studies have highlighted the benefit of using programs that help to develop constructive commitment and sense of belonging to families, schools, and communities. This is performed through the development of social skills and competences that allow them to communicate effectively, make better decisions, build better self-esteem, and have a greater sense of personal responsibility (48, 49). Additionally, interventions that promote positive relationships between teachers and students and a sense of belonging may also help to promote other healthy behaviors (50).

This study had some limitations. It is a cross-sectional study; therefore, we cannot rule out reverse causality in the associations we found. A longitudinal design may help to understand the relation in time between school membership and substance use (46). There may be other potential confounders that were not measured, such as academic performance or peer influence, that may explain some residual confounds. Additionally, all data were collected using self-reported questionnaires, which may have some reporting bias. Although this is a large and representative sample of secondary students attending state founded schools in Santiago of Chile, it may not represent younger or older students in those schools nor students attending subsidized and private schools in Santiago or other cities in Chile. Finally, the 30-day usage of cigarette, alcohol, and cannabis as outcome measures may have limitations to fully understand the experience of substance use among adolescents; however, this is a recommended time interval used in school surveys (23), and the dichotomous approach is widely used, allowing us to compare our findings with other studies (51, 52).

## Conclusion

This is the first study in Latin America exploring the association between school membership and substance use among secondary students. We found that those students having a high sense of school membership had a lower risk of smoking, drinking, and cannabis use compared with students who had low-school membership. We also found that part of this effect is mediated by the presence of depressive symptoms and poor self-concept. These results provide support to test interventions aimed at increasing the sense of membership to prevent substance use among adolescents.

## Availability of Data and Materials

The data can be accessed from the corresponding author through the following address jgaete@uandes.cl, after all main results generated from these data, according to the Funding, are published. The data will be accessed for research purpose and this is because during the ethical clearance process we agree with the Ethical Committee of University of Chile to keep the confidentiality of the data set.

## Ethics Statement

This study was approved by the local committee of ethics at the Hospital Clínico Universidad de Chile (No. 179; June 30, 2008), and informed consent from parents or main caregivers and assent from students were required to participate.

## Author Contributions

RA and JG conceived and designed the study. JG, RF, and GR supervised the collection of data. JG and RA analyzed and interpreted the data, and produced the drafting of the manuscripts. All authors provided a critical revision of the manuscript.

## Conflict of Interest Statement

The authors declare that the research was conducted in the absence of any commercial or financial relationships that could be construed as a potential conflict of interest.

## Abbreviations

PSSM, psychological sense of school membership scale; BDI-II, Beck Depressive Inventory, second version; RCADS, revised child anxiety and depression scale; CATS, children’s automatic thoughts scale; SPSI-R, social problem-solving inventory revised.
